# *Termitotrox venus* sp. n. (Coleoptera, Scarabaeidae), a new blind, flightless termitophilous scarab from Cambodia

**DOI:** 10.3897/zookeys.513.9958

**Published:** 2015-07-15

**Authors:** Showtaro Kakizoe, Munetoshi Maruyama

**Affiliations:** 1 Laboratory of Ecological Science, Department of Biology, Faculty of Sciences, Kyushu University, Hakozaki 6-10-1, Fukuoka, 812-8581 Japan; 2 The Kyushu University Museum, Hakozaki 6-10-1, Fukuoka, 812-8581 Japan

**Keywords:** Termitophily, Termitotroginae, Termitotrogini, Isoptera, Termitidae, Macrotermitinae, *Macrotermes*, new species, Indo-Chinese subregion, mouthparts

## Abstract

*Termitotrox venus* **sp. n.** is described from Cambodia and represents the second discovery of *Termitotrox* Reichensperger, 1915 from the Indo-Chinese subregion of the Indomalayan region. Most of the type series was collected from refuse dumps in fungus garden cells of Macrotermes cf. gilvus (Hagen, 1858). *Macrotermes* Holmgren, 1910 was previously an unknown host of *Termitotrox* species. The new species is easily distinguished from all known congeners by having wing-shaped trichomes on the elytra and the larger body size, at 2.5 mm in length. We also describe the mouthparts to complement the description of the genus *Termitotrox*.

## Introduction

Members of the genus *Termitotrox* Reichensperger, 1915 are blind, flightless termitophilous scarabs associated with the fungus-growing termite genera *Protermes* Holmgren, 1910, *Odontotermes* Holmgren, 1912 or *Hypotermes* Holmgren, 1917 (Isoptera, Termitidae, Macrotermitinae). The genus was previously known from the Ethiopian region (eight species) and the Indian subregion (two species) of the Indomalayan region (Krikken 2008), until the discovery of *Termitotrox cupido* Maruyama, 2012 from Cambodia, representing the first species of *Termitotrox* from the Indo-Chinese subregion of the Indomalayan region (Maruyama 2012a). Recently, we collected another undescribed species of *Termitotrox* in Cambodia from fungus garden cells of *Macrotermes* Holmgren, 1910 (also Macrotermitinae) – a previously unknown host of *Termitotrox*. This is the second discovery of the genus in the Indo-Chinese subregion of the Indomalayan region. In this paper the new species is described and biological information about it is provided.

## Materials and methods

In August 2014, we examined fungus gardens of the termite genera *Macrotermes*, *Microtermes* Wasmann, 1902, *Odontotermes* and *Hypotermes* in Siem Reap, Cambodia. After examining more than 300 fungus gardens, we found 8 *Termitotrox* beetles from fungus garden cells of Macrotermes cf. gilvus (Hagen, 1858) and *Hypotermes makhamensis* Ahmad, 1965, seven specimens in seven cells of three colonies of Macrotermes cf. gilvus and one specimen from one cell of *Hypotermes makhamensis*. The beetles were put in a killing tube (35 ml) with tissue paper and ethyl acetate; a day later they were removed from the tube and kept in 80% ethanol. All specimens were dried and mounted for morphological observation. Dissected genitalia and mouthparts were mounted in Euparal on a small glass plate (10×5 mm), and subsequently glued onto a paper card (6×5 mm) and pinned under the respective specimen (Maruyama 2004). A permanent mount of mouthparts was also made. Specimen photographs were taken using a Canon EOS 60D with a Canon MP-E 65 mm 1–5× macro lens and Kenko extension tubes and stacked using CombineZP software. Images of living beetle were taken using a Canon EOS 7D with a EF 100mm F2.8L Macro lens and Kenko extension tubes. Terminology of the species description follows Krikken (2008). All measurements in the paper are given in millimeters as follows: minimum length – maximum length (mean ± SD). The type series is deposited in Maruyama collection in the Kyushu University Museum, Fukuoka, Japan.

## Taxonomy

### Genus Termitotrox

Taxon classificationAnimaliaColeopteraScarabaeidae

Reichensperger

Figs 5–8

Termitotrox Reichensperger 1915: 16 (type species: *Termitotrox consobrinus* Reichensperger, 1915, by monotypy).
Aphodiocopris Arrow 1920: 432 (type species: *Aphodiocopris minutus* Arrow, 1920, by monotypy).

#### Additional description.

Maxillae (Fig. 5) small; mala toothed distally; basistipes and cardo with long setae on lateral side. Maxillary palpus 4-segmented and well developed; segment I small, bent outwards; segment II about 2 times as long as segment I; segment III small, only slightly longer and broader than segment I, slightly bent inwards; segment IV large, approximately twice as long as segment II; numerous digitiform sensillae present on ventrolateral side of proximal half of segment IV. Labial palpus strongly reduced. Mandibles (Figs 6, 7) asymmetrical, pointed apically, numerous serrate ridges on molar surface. Epipharynx (Fig. 8) with anterior margin feebly bisinuate, epitorma almost indistinct, pedia almost glabrous, chaetoparinae very strong and elongate.

#### Comments.

See Krikken (2008) for generic review. No detailed mouthparts description has previously been provided for *Termitotrox*. Although this additional description is based on only two species, *Termitotrox cupido* and *Termitotrox venus*, the other members of *Termitotrox* are expected to share the same or similar character states based on their overall similarity of external morphology.

### Termitotrox venus

Taxon classificationAnimaliaColeopteraScarabaeidae

Kakizoe & Maruyama sp. n.

http://zoobank.org/8174A036-4FCC-4957-B788-2541A6CA13D1

Figs 1–4 , 5–10 , 11–13

#### Type materials.

Holotype, male, deposited in Maruyama collection in the Kyushu University Museum: 1.0 km south of Angkor Wat, Angkor, Siem Reap, Cambodia, 22 VIII 2014, S. Kakizoe leg. Paratypes, deposited in Maruyama collection in the Kyushu University Museum: 1 female, 1.6 km southwest of Angkor Wat, Angkor, Siem Reap, Cambodia, 20 VIII 2014, S. Kakizoe leg.; 1 male, 1.0 km south of Angkor Wat, Angkor, Siem Reap, Cambodia, 24 VIII 2014, S. Kakizoe leg.; 2 males, 2 females, 0.77 km east of Angkor Wat, Angkor, Siem Reap, Cambodia, 24 VIII 2014, M. Maruyama & S. Kakizoe leg. (In fungus garden cells of Macrotermes cf. gilvus); 1 female, 1.7 km east of Neak Pean, Angkor, Siem Reap, Cambodia, 25 VIII 2014, S. Kakizoe leg. (In fungus garden cell of *Hypotermes makhamensis*).

#### Distribution.

Northwestern Cambodia.

#### Etymology.

*Venus* is the goddess of fertility, beauty and love in ancient Roman mythology and is often illustrated together with *Cupido*. The new species was found in the area where *Termitotrox cupido* was also found. Therefore, this species is named *Venus*. Noun in apposition.

#### Diagnosis.

This species is similar to *Termitotrox cupido* in having the wing-shaped trichomes on the elytra but easily distinguished from it by the larger body and the development of the pronotal basomedian section and the elytral median projection.

#### Description of holotype male.

**Figures 1–4.**
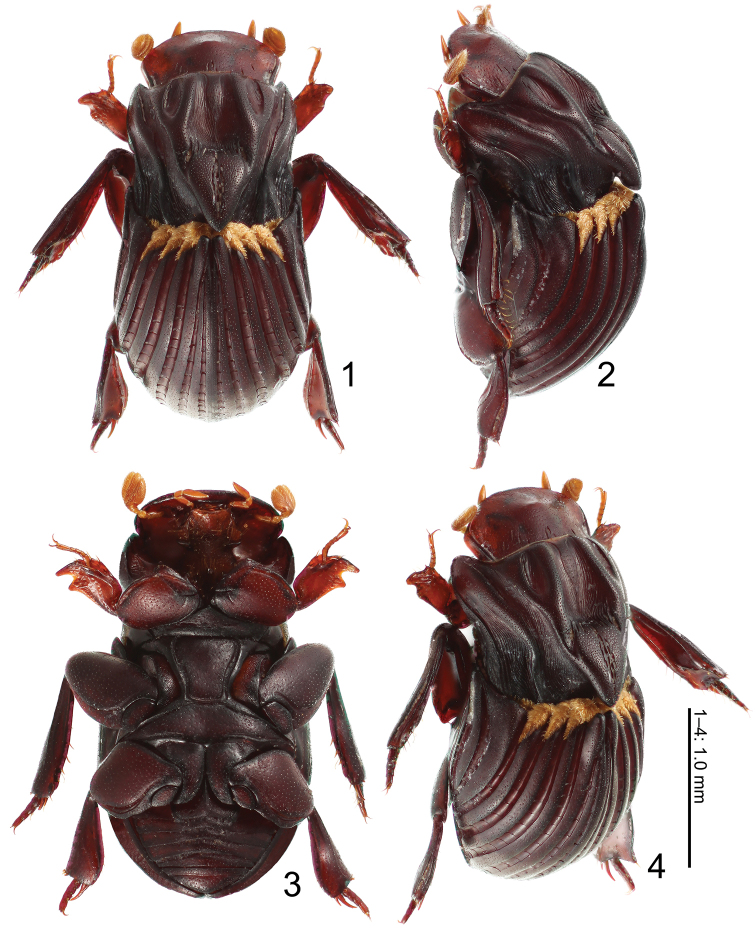
Male habitus of *Termitotrox venus* sp. n. **1** dorsal view **2** lateral view **3** ventral view **4** antero-lateral view

General color (Figs 1–4, 11–13) uniformly dark brown, matt, body length 2.46 mm. ***Head*** (Figs 1–4). Surface generally evenly convex, only with a slight callosity at clypeofrontal transition. Lateral margin of head entirely, finely marginate. Clypeal outline evenly rounded. Clypeofrons brown, glabrous, distinctly, moderately punctate, and 9 elongate deep punctures. Genal tip obtusely angular (in dorsal view); genal surface depressed with deep groove medially. Antennae (Fig. 3) yellowish brown with setate club. ***Prothorax*** (Figs 1–4). Prothorax dark brown, narrower than elytra, sides (in dorsal view) evenly rounded over anterior half. Anterolateral lobe rounded, edge slightly projecting downward (forming side of anterolateral propectoral ridge). Pronotal sides steeply declivous. Posterolateral section of pronotum rounded. Base of pronotum evenly rounded, immarginate; basolateral area with 1 fine ridge and numerous grooves around base. Pronotal surface glabrous. Costae densely punctate, intercostal sulci with distinctly wrinkled. Discal depression deep; surface, apart from some local micropunctation, smooth. Pronotal pattern of longitudinal costae as follows: median costa broad, becoming indistinct around apical 1/5; basomedian section triangular, surface deplanate, flattened except a longitudinal wrinkled furrow at middle. Central depression posterolaterally delimited by depressed area of paramedian costa. Paramedian costa broad, distinct, continuing to about 2/3 of pronotal length. Sublateral costa anteriorly broad, distinct, tapering posteriad to about 1/5 of pronotal length, reaching paramedian costa. Lateral costa anteriorly broad, distinct, extending from anterolateral lobe caudad, tapering to base of pronotum. Marginal costa posteriorly broad, ending at depressed basolateral area. Anterolateral part of propectus deeply excavate. Preprosternal apophysis distinct, with several setae. Propectus glabrous, brown. Posterolateral area of propectus with some ridges and grooves. Postprosternal surface with small, shallow, median impression. ***Elytra*** (Figs 1–2, 4). Semi-elliptical, strongly convex dorsally, as high as pronotum, dark brown, matt, with 7 interstrial costae and intervening striae, and with short adpressed trichomes at base of costae 2–5 forming wing-shaped patches. Humeral and apical elytral umbones absent; apicosutural edge nearly rectangular, strongly protruding above. Epipleuron wide. Elytral striae distinct, deeply impressed, with transverse weak costae from base to apex forming quadrate cells; striae 1 and 2 reaching basal half. Discal interstrial costae broadly trapezoidal (in cross-section), surface with dense, scattered punctures. Elytral pattern of interstrial costae as follows: costa 1 (next to suture) narrow, almost rectilinear; costa 2 tapering in front, stopping at basal half. Costa 3 complete, strongly developed, stoutly protruding in front to form median projection. Costae 4–8 complete, strongly developed. Costae 9 and 10 apparently fused together. ***Mesosternum*** (Fig. 3). Transverse mesometasternal groove between posterior edges of mesocoxae distinct, straight, not completely reaching mesocoxae. Mesosternum with fine peridiscal grooves arising from this transverse groove and two diagonal grooves, except in front; mesosternal surface dark brown, glabrous, flattened, moderately micropunctate. ***Metasternum*** (Fig. 3). Metasternum with very shallow median impression, glabrous, and with fine perimarginal groove all around; dark brown. ***Abdomen*** (Fig. 3). Venter with 5 visible fairly sclerotized sternites, all dark brown, matt, glabrous, without grooves, sparsely micropunctate. Pygidium dark brown, glabrous, base broadly margined; surface generally convex, densely micropunctate. ***Legs*** (Figs 1–4). Procoxa protuberant. Profemur brown, underside glabrous, densely micropunctate; outline broadly elliptical, emarginate distally. Protibia pale brown, broad, with sparse short setae, microsculpture poorly pronounced; shape strongly complanate with 2 external denticles, no basal serration; apex oblique-sinuate, transverse, with distinct apico-internal spine; internal side strongly dilated from slender base. Protarsus twice longer than width of tibial apex, slender, yellowish brown; segment 1 inserted in fine groove, as long as segments 2–4 combined. Mesocoxa dark brown, widely separated, slightly divergent anteriad. Mesofemur dark brown, broadly elliptic in outline, distally emarginate, surface moderately micropunctate, glabrous. Mesotibia dark brown, with several setae, broad, dilated near base, nearly parallel-sided from apex, edges entire; tibial apex deeply emarginate, with pair of acuminate apico-internal spurs, external one long, slightly curved, internal one short, straight; upper side of mesotibia with fine longitudinal ridge near outer edge, weak costa at basal half, underside with fine sinuate ridge from base to apico-internal section; with long setae around apical quarter. Metatibia similar to mesotibia, but gently dilated apicad, with apex shallowly emarginate. Meso- and metatarsi dark brown, compacted-complanate, segments 1–4 short. Length of outer apical spur of metatibia 1/4 of metatibia, reaching base of tarsal segment 5. Aedeagus (Figs 9, 10).

**Figures 5–10.**
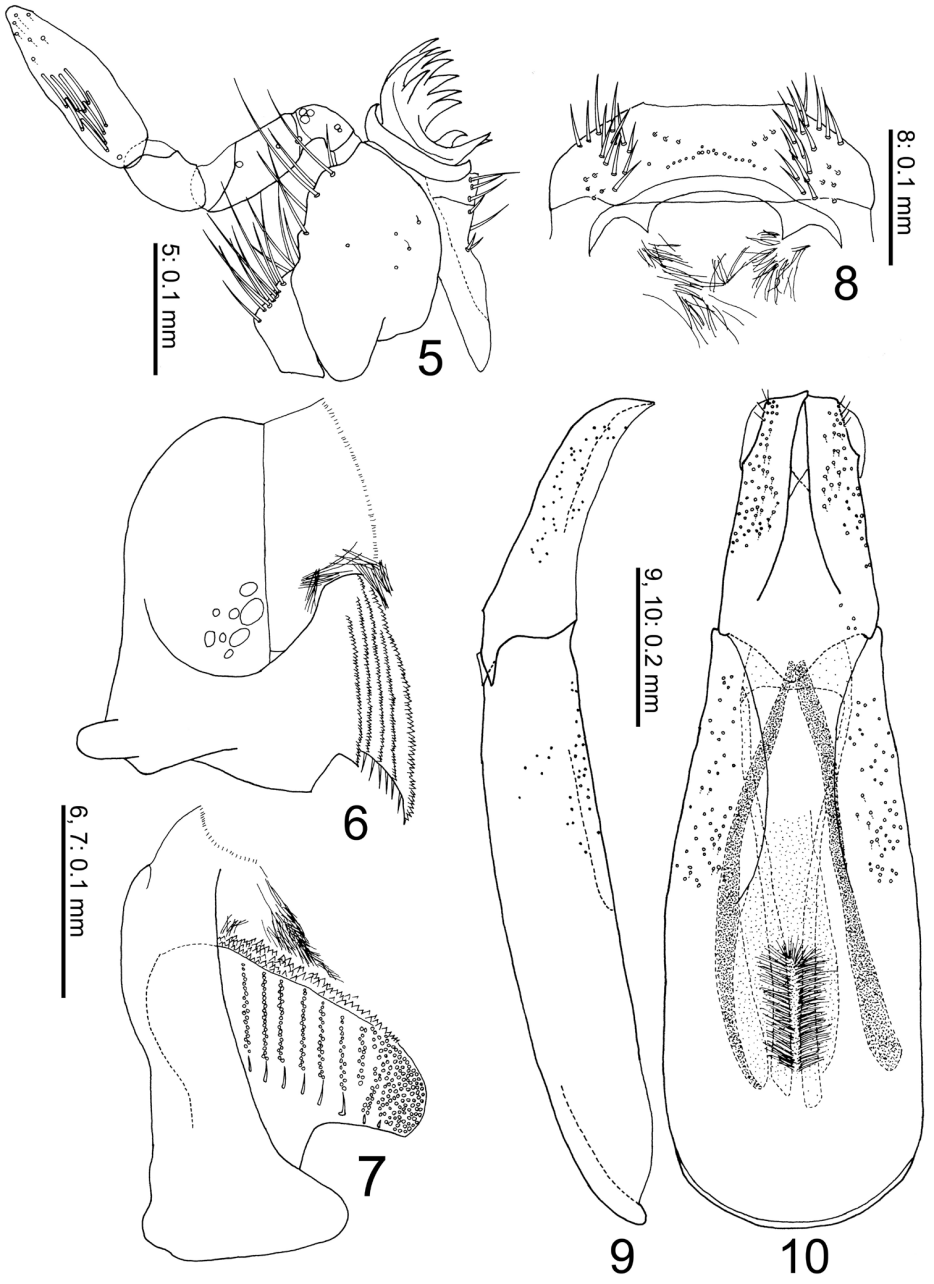
Body parts of *Termitotrox venus* sp. n. **5** maxilla (without cardo) **6, 7** right mandible, in ventral and lateral view **8** epipharynx **9, 10** aedeagus in lateral and dorsal views.

**Figures 11–13.**
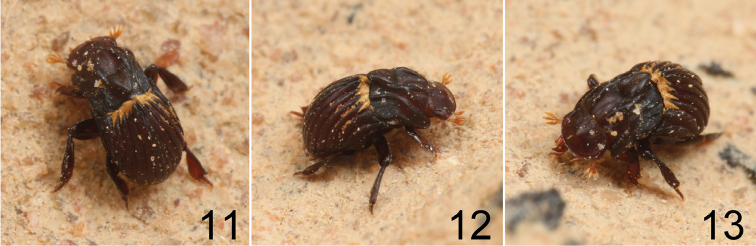
Living *Termitotrox venus* sp. n. walking on a wall of the host termite nest inside.

#### Female.

No significant sexual dimorphism is detected.

#### Measurements.

Body length 2.26–2.70 (2.48±0.124); maximum width of head 0.84–0.93 (0.88±0.031); median dorsal length of pronotum 0.92–1.14 (1.01±0.064), maximum width 0.98–1.12 (1.04±0.053); sutural length of elytra 1.12–1.53 (1.35±0.115), maximum width 1.14–1.34 (1.24±0.067). *N* = 7.

#### Symbiotic host.

Macrotermes cf. gilvus (see Discussion).

#### Remarks.

Male aedeagus size ratio is the same rate as *Termitotrox cupido*, i.e. 44% of body length.

## Discussion

**Termite association.** Of the eight *Termitotrox venus* beetles recovered from fungus garden cells of Macrotermes cf. gilvus (Figs 11–13), seven were found on or inside the refuse dumps. The refuse dumps do not contain any fungal carpophores. The beetles appear camouflaged inside the refuse and move slowly, so they are difficult to collect. Only one specimen was found on the wall of fungus garden cell of *Hypotermes makhamensis* (Figs 11–13) despite more than 200 fungus garden cells of this termite were examined; this is probably accidental (it may be caused by the underground connection of the colonies of the two termite species ). Therefore, we think that the true host of *Termitotrox venus* is Macrotermes cf. gilvus.

All other known termitotrogines are associated with either *Protermes*, *Odontotermes* or *Hypotermes* (Krikken 2008; Maruyama 2012a). A phylogenetic analysis of fungus-growing termites revealed that these three genera form a monophyletic group, with *Odontotermes* being paraphyletic with respect to *Hypotermes*; however, *Macrotermes* did not group with this clade, and is instead only distantly related (Aanen and Eggleton 2005). In contrast, *Termitotrox venus* and *Termitotrox cupido* have a clear synapomorphy in the wing-shaped trichomes on the elytra, so these species are apparently closely related to each other. Therefore, the host relationship between species of *Termitotrox* and genera of Macrotermitinae is unlikely to have arisen via co-cladogenesis. This type of relationship between termite hosts and termitotrogine scarabs is similar to that observed in Corythoderini. Corythoderines are also known to be associated with *Odontotermes* and *Macrotermes* (Tangelder and Krikken 1982; Bordat and Moretto 2010; Maruyama 2012b). This capacity to utilize phylogenetically unrelated hosts suggests that perhaps both *Macrotermes* and the group formed by *Protermes*+*Odontotermes*+*Hypotermes* produce similar nest odors, which are targeted by termitophilous scarabs in search of host colonies.

The pronotal basomedian section and the elytral median projection of *Termitotrox venus* form a structure (Figs 2, 4) similar to that seen in *Eocorythoderus incredibilis* Maruyama, 2012 (and, to a lesser extent in *Termitotrox cupido*), which was also found in a Macrotermes cf. gilvus nest in Siem Reap. Maruyama (2012b) revealed that this structure functions as a handle that allows the termite to grip the beetle and carry it. We did not observe *Termitotrox venus* being carried by worker termites during our survey, but this structure is probably used for the same behavior. In addition, the number of damaged specimens (broken legs, tibia or tarsi) of this species was lower than that of *Eocorythoderus incredibilis* (damaged/undamaged: *Termitotrox venus* 2/8, *Eocorythoderus incredibilis* 5/10, based on the type series from Maruyama (2012b)), hence, perhaps, *Termitotrox venus* could be mostly a synoekete (ignored by the hosts) except during certain periods, such as the movement of the host colony.

Using Wasmannian terminology (Wasmann 1894), Vårdal and Forshage (2010) suggested Termitotrogini may be synechthrans (treated with hostility by the hosts) because of the defensive morphology of the species known at that time. However, at least *Termitotrox venus* and *Termitotrox cupido* seem to be mainly synoeketes because both species appeared to be ignored by termites in the field. Based on both morphology and field observations, Corythoderini were proposed to be symphilic (Vårdal and Forshage 2010). Although symphilic behavior was recorded for *Eocorythoderus incredibilis* (Maruyama 2012b), the rate of specimen damage is nevertheless high, and this species has, in overall, a more defensive morphology compared to the other species of Corythoderini. Hence, the biology of *Eocorythoderus incredibilis* may vary from persecuted (synechthran) to integrated (symphile). On the other hand, *Termitotrox venus* may be a largely ignored (synoekete) but based on the morphology similar to that of *Eocorythoderus incredibilis* (trichomes, carrying “handle”), may at times exhibit symphilic behavior. The discoveries of these new combinations of lifestyles in termitophilous beetles require a more flexible framework than that proposed by Wasmann (1894).

**Size difference.** *Termitotrox venus* is larger than *Termitotrox cupido*, and the beetle size seems to be correlated with the body size of the primary host of each of these species. Therefore, inquiline size may be affected by host size — a relationship paralleling that seen between termitophilous Staphylinidae and their hosts (Maruyama, personal observation).

## Supplementary Material

XML Treatment for Termitotrox

XML Treatment for Termitotrox venus
